# Psycho-education with problem solving (PEPS) therapy for adults with personality disorder: A pragmatic multi-site community-based randomised clinical trial

**DOI:** 10.1186/1745-6215-12-198

**Published:** 2011-08-24

**Authors:** Mary McMurran, Mike J Crawford, Joseph G Reilly, Paul McCrone, Paul Moran, Hywel Williams, Clive E Adams, Conor Duggan, Juan Delport, Diane Whitham, Florence Day

**Affiliations:** 1 Institute of Mental Health, University of Nottingham, Sir Colin Campbell Building, Triumph Road, Nottingham NG7 2TU, UK; 2 Clinical Trials Unit, Nottingham Health Science Partners, C Floor, South Block, Queen's Medical Centre, Nottingham NG7 2UH, UK; 3 Centre for Mental Health, Imperial College London, 37 Claybrook Road, London W6 8LN, UK; 4 Wolfson Research Institute, University of Durham, Queen's Campus, Stockton on Tees TS17 6BH, UK; 5 King's College London (Institute of Psychiatry), King's College London, London SE5 8AF, UK; 6 Psychological Therapies Centre, St Tydfil's Hospital, Merthyr Tydfil, CF47 0SJ, UK

## Abstract

**Background:**

Impairment in social functioning is a key component of personality disorder. Therefore psycho-education and problem solving (PEPS) therapy may benefit people with this disorder. Psycho-education aims to educate, build rapport, and motivate people for problem solving therapy. Problem solving therapy aims to help clients solve interpersonal problems positively and rationally, thereby improving social functioning and reducing distress. PEPS therapy has been evaluated with community adults with personality disorder in an exploratory trial. At the end of treatment, compared to a wait-list control group, those treated with PEPS therapy showed better social functioning, as measured by the Social Functioning Questionnaire (SFQ). A definitive evaluation is now being conducted to determine whether PEPS therapy is a clinically and cost-effective treatment for people with personality disorder

**Methods:**

This is a pragmatic, two-arm, multi-centre, parallel, randomised controlled clinical trial. The target population is community-dwelling adults with one or more personality disorder, as identified by the International Personality Disorder Examination (IPDE). Inclusion criteria are: Living in the community (including residential or supported care settings); presence of one or more personality disorder; aged 18 or over; proficiency in spoken English; capacity to provide informed consent. Exclusion criteria are: Primary diagnosis of a functional psychosis; insufficient degree of literacy, comprehension or attention to be able to engage in trial therapy and assessments; currently engaged in a specific programme of psychological treatment for personality disorder or likely to start such treatment during the trial period; currently enrolled in any other trial. Suitable participants are randomly allocated to PEPS therapy plus treatment as usual (TAU) or TAU only. We aim to recruit 340 men and women. The primary outcome is social functioning as measured by the SFQ. A reduction (i.e., an improvement) of 2 points or more on the SFQ at follow-up 72 weeks post-randomisation is our pre-specified index of clinically significant change. Secondary outcomes include a reduction of unscheduled service usage and an increase in scheduled service usage; improved quality of life; and a reduction in mental distress.

**Discussion:**

PEPS therapy has potential as an economical, accessible, and acceptable intervention for people with personality disorder. The results from this randomised controlled trial will tell us if PEPS therapy is effective and cost-effective. If so, then it will be a useful treatment for inclusion in a broader menu of treatment options for this group of service users.

**Trial Registration:**

International Standard Randomised Controlled Trial Number - ISRCTN70660936

## Background

Personality disorder (PD) is a prevalent mental disorder, affecting over 4% of the general population [1]. People with personality disorder suffer high levels of distress, suicide, self-harm, addiction, family breakdown, and social exclusion. Rendu et al. [2] assessed the costs of treating people with PD in primary care as £3,094 per annum, almost twice the costs of people without PD. A prevalence of PD of almost 5% equates to around 3 million people in the UK, costing around £40 million per annum extra to regular care in primary care alone (at 2002 costs). Furthermore, people with PD place considerable demands upon a range of services, including emergency departments, social services, and the criminal justice system. For those who enter outpatient specialist mental health services, treatment costs are around £135 per treatment session [3], and treatment for people with PD is often of long duration. People with PD who offend may enter medium secure services, where the cost is around £176,000 per person per year [3]. If a relatively brief and effective treatment can be offered to a broad spectrum of people with PD dwelling in the community, this will have the potential to improve the lives of people with PD, improve access to services, and also reduce health and non-health service costs.

Systematic reviews of outcomes of psychological treatments for people with PD [4-7] have identified few randomised controlled trials (RCTs). The majority of these studies are underpowered, most have multiple outcome measures, and only about one-third measure social functioning, which is agreed to be the most significant clinical problem for this group of patients. We plan to conduct a well-designed and adequately-powered study of one promising intervention, namely psycho-education combined with problem solving (PEPS) therapy, which has become a popular way of working. Enthusiasm for its use does not obviate the need for a rigorous evaluation of the effectiveness of PEPS therapy; indeed, if it is widely used then evidence of effectiveness - or otherwise - is crucial.

Social problem solving is the process by which an individual attempts to identify or discover solutions to specific problems encountered in everyday living [8]. Good social problem solving skills help people to cope with life's stressors, particularly those with an interpersonal component [9]. Impairment in social functioning is a key component of PD [10-12], hence the emphasis on social functioning that underpins problem solving therapy is highly relevant to the treatment of PD. Using the Social Problem Solving Inventory-Revised (SPSI-R) [13], we have found that people with PD, both community adults presenting for treatment and detained offenders with PD, report greater impairment on all SPSI-R scales compared to a sample of mature students [9]. This information suggests that social problem solving therapy may benefit people with PD.

Meta-analyses have shown problem solving therapy to be effective in improving a range of mental and physical health problems [14-16]. This therapy has not been evaluated with people with PD, nonetheless it is suited to this group because the focus is upon improving social functioning and reducing personal distress. These outcomes are considered to be of paramount importance in the treatment of PD [17]. Furthermore, the aim in therapy is to help people recognise both their strengths and limitations and work with these to learn new skills that will enable them to cope more effectively with life's problems. People with PD have different trait profiles and different problem solving deficits [18], and problem solving therapy is sufficiently flexible to accommodate these differences. It helps clients to adopt a more realistically positive orientation to problem solving, cope better with the negative emotions that hinder effective problem solving, develop a positive problem orientation, and adopt a rational problem solving style that will lead to outcomes that improve social functioning and reduce distress.

Engaging people with PD in treatment is a major challenge [19,20]. The social problem solving approach enhances engagement by offering an accessible framework for change (i.e., the approach appeals to common sense), supporting people in the experience of successful problem solving (i.e., increasing the likelihood of reinforcement of the developing skills), and encouraging independence rather than reliance on therapy (i.e., promoting self-efficacy). Furthermore, PEPS therapy has a preliminary psycho-education component which aims to educate, build rapport, and motivate people for problem solving therapy [21]. Personality disorders and their impact are discussed in a collaborative dialogue and problems that may be worked upon in group sessions are identified.

PEPS therapy has been evaluated with community adults with PD in a Phase 2 exploratory trial [22]. At the end of treatment, compared to a wait-list control group, those treated with PEPS therapy showed better social functioning, as measured by the Social Functioning Questionnaire [23]. All aspects of social problem solving improved over the course of PEPS therapy, and, after controlling for baseline level of social functioning, the most important predictor of improvement in social functioning was a reduction in negative problem orientation, i.e., people felt less threatened by problems and more confident in their ability to solve them [24].

This exploratory study has been identified as important in four ways [25,26]. First, the intervention was brief and hence is likely to be more acceptable to many patients than lengthier interventions; this decreases the likelihood of drop-out and may also be more acceptable to services with limited resources. Second, PEPS therapy was delivered in clinical settings, hence its likely effectiveness in everyday practice was indicated. Third, PEPS therapy was offered to people with any PD or combination of PDs, so it was inclusive rather than exclusive. Fourth, PEPS therapy was delivered by non-specialist staff, hence it would be possible to deliver it relatively cheaply.

Overall, PEPS therapy has the potential to contribute to the UK National Health Service's (NHS) QUIPP agenda (Quality, Improvement, Productivity and Prevention): it is a brief, innovative intervention in which staff can easily be trained and which could be made widely available to people with personality disorder at a stage where prevention of deterioration is possible. However, a definitive evaluation now needs to be conducted. A trial will permit investigation of aspects of the therapy about which there is currently no information. First, a longer-term follow-up is necessary to provide information about the sustainability of gains made during treatment. Second, a cost-effectiveness evaluation of the intervention is required. Third, there is a need to gather information about the respective contributions of psycho-education and social problem solving therapy. We are now conducting a Phase 3 definitive randomised controlled trial to consolidate and extend our knowledge about the effectiveness of PEPS therapy for people with personality disorder (see http://www.peps-trial.co.uk). If improvements in social functioning can be definitively shown to result from therapy and be sustained over time (72 weeks post-randomisation), then this relatively brief intervention could be used more widely across the NHS.

### Study Aims

1. To conduct a randomised controlled trial to evaluate the effectiveness of PEPS therapy compared with treatment as usual in improving social functioning in community adults with personality disorder.

2. To assess the costs and cost-effectiveness of PEPS therapy compared with treatment as usual.

3. To examine intermediate change, specifically the impact of psycho-education on the therapeutic relationship, and the impact of social problem solving therapy on social problem solving skills.

5. To conduct a qualitative investigation of the application of PEPS therapy in practice to identify the views of participants.

### Study Hypotheses

Our primary hypothesis is that, compared with those in treatment-as-usual, those in PEPS therapy will show a greater improvement in social functioning post-therapy and at follow-up 72 weeks post-randomisation. Secondary hypotheses are that, compared with those in treatment-as-usual, those in PEPS therapy will show the following changes both immediately after therapy and at follow-up: (a) a greater reduction in receipt of unscheduled services; (b) a greater increase in receipt of scheduled services; (c) a greater improvement in quality of life; (d) a greater improvement in referrers' ratings of functioning; (e) a greater reduction in anxiety and depression; and (f) improvement on client-ratings of self-identified three key problems. Regarding intermediate changes, we hypothesise that, compared with those in treatment-as-usual, those in PEPS therapy will show: (a) better therapist alliance after psycho-education; and (b) a greater improvement in social problem solving at end of therapy. We also expect PEPS therapy to show an acceptable level of cost-effectiveness, based on thresholds that appear to guide National Institute for Clinical Excellence (NICE) recommendations.

## Method

### Design

This is a pragmatic, two-arm, multi-centre, parallel, randomised controlled clinical trial.

### Ethics

Approval for the research was given by the South East Wales Research Ethics Committee - Panel C (Ref: 09/WSE03/48) and from the Research and Development (R&D) departments of the participating NHS Trusts: Central and North West London NHS Foundation Trust (24195/LNW); Cwm Taf Health Board (CT/039/09); Tees, Esk and Wear Valleys NHS Foundation Trust (24195/CDTV).

### Participants

The target population is community-dwelling adults with one or more PD diagnoses, as identified by the International Personality Disorder Examination [27]. Inclusion criteria are: Living in the community (including residential or supported care settings); presence of one or more personality disorder; aged 18 or over; proficiency in spoken English, assessed if necessary by the Basic Skills Agency 'Fast Track 20 Questions' [28]; capacity to provide informed consent. Exclusion criteria are: Primary diagnosis of functional psychosis; insufficient degree of literacy, comprehension or attention to be able to engage in trial therapy and assessments; currently engaged in a specific programme of psychological treatment for personality disorder or likely to start such treatment during the trial period; currently enrolled in any other trial.

### Sample size

The sample size was calculated on the basis of the primary hypothesis. In the exploratory study [22], those referred to PEPS had a greater improvement in social functioning at 6 month follow-up equivalent to 1.05 points on the SFQ. However, a number of people received PEPS who were not included in the trial (e.g., the wait-list control) and, for this larger sample (N = 93), the mean pre-post- treatment difference was 1.79 (pre-treatment mean = 13.85, SD = 4.21; post-treatment mean = 12.06, SD = 4.21). (Note: a lower SFQ score is more desirable). This difference of almost 2 points accords with other evidence that this is a clinically significant and important difference [29]. A reduction of 2 points or more on the SFQ at 1 year follow-up in an RCT of cognitive behaviour therapy in health anxiety was associated with a halving of secondary care appointments (1.24.vs 0.65), a clinically significant reduction in the Hospital Anxiety and Depression Scale (HADS [30]) Anxiety score of 2.5 (9.9 vs 7.45) and a reduction in health anxiety (the main outcome) of 5.6 points (17.8 vs 12.2) (11 is a normal population score and 18 is pathological) [29]. These findings suggest that improvements in social functioning may accrue over 1 year, hence we expect to find a greater magnitude of response at the 72 week follow-up than we did in the exploratory trial. Therefore, we have powered this trial to be able to detect a difference in SFQ score of 2 points. SFQ standard deviations vary between treatment, control, and the wait-list samples, ranging from 3.78 to 4.53. We have based our sample size estimate on the most conservative (i.e., largest) SD. To detect a mean difference in SFQ score of 2 point (SD = 4.53) at 72 weeks with a two-sided significance level of 1% and power of 80% with equal allocation to two arms would require 120 patients in each arm of the trial. To allow for 30% drop out, 170 will be recruited per arm, i.e., 340 in total. We have considered the need to take clustering effects by therapy group into account. In this study, as in the pilot, participants are individually randomised to the treatment arms, and the observed SD of the response in the intervention arm automatically includes the effect of the clustering by therapist. The analysis will take account of this by using a hierarchical model to allow explicit estimation of the between therapy group variance.

### Recruitment

**Figure 1:**
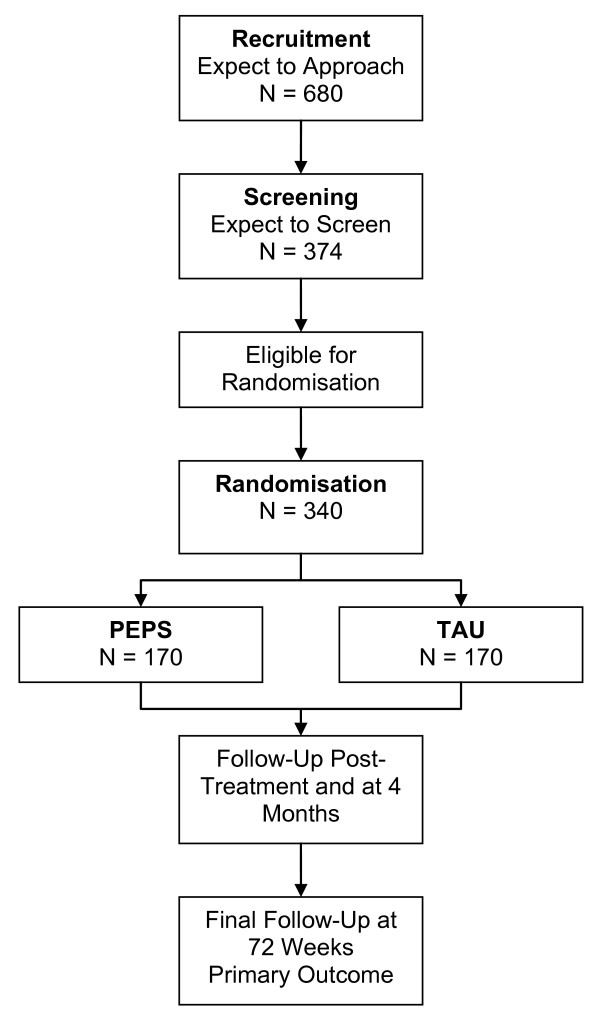
**Recruitment Flow Diagram**.

To recruit the required number (N = 340), we estimated that we need to approach twice as many people (N = 680). This estimate is based upon the exploratory trial, in which 464 potential participants were approached to be invited to participate; of these, 316 (68%) volunteered to participate; 255 (55%) turned up for the assessment interview; 241 (52%) met the criteria and hence were available to be randomised. A recruitment flow chart is presented in Figure 1.

Three sites are participating in the trial. Each of the three sites needs to refer approximately 230 people to the project over 26 months (approximately 106 per year). Based on information about the sites, this was considered to be feasible. In West London, the two local Mental Health Trusts from which we are recruiting serve a population of 1.7 million people, with potentially 74,800 people with personality disorder. In 2007, one of the five boroughs received 80 referrals of people with personality disorder who were motivated to receive psychological treatment for their disorder. New specialist services were planned for 2009 in two of the other boroughs and similar rates of referral to these new services were expected. Cwm Taf NHS Trust serves a population of 330,000 with potentially 14,520 people with personality disorder. In 2007, there were 4,173 referrals per annum across two Community Mental Health Teams (CMHTs) and two crisis teams. A retrospective audit of randomly selected current case notes (N = 109) found that between 8 and13% of people seen by one CMHT met the DSM-IV criteria for borderline personality disorder. Therefore, an estimated 333 to 542 of the people annually referred to mental health services were expected to meet the diagnostic criteria for borderline personality disorder. Assuming that this figure represents between 30% and 60% of the population with personality disorders, it can be estimated that an additional number of between 133 and 217 people meet the criteria of a personality disorder other than borderline. Tees, Esk and Wear Valley NHS Foundation Trust serves a total population of 1.4 million across County Durham, the Tees Valley and North East Yorkshire. One existing advanced practitioner service for personality disorder covering a population of 100,000 received approximately 50 referrals per year, primarily from CMHTs, of individuals likely to benefit from psychological intervention. This service was planning to expand its remit to the larger area of North Durham (300,000 population) and Teesside (500,000 population), hence it was expected that an expanded service would receive up to 400 referrals annually for all personality disorders by the study start date, requiring a referral rate of the study of less than 25% to meet the target.

### Randomisation and blinding

Randomisation is based on a computer generated pseudo-random code using random permuted blocks of randomly varying size, created by the Nottingham Clinical Trials Unit (CTU) in accordance with their standard operating procedure and held on a secure server. The randomisation is stratified by recruiting centre and sex of participant. Access to the sequence is confined to the Trial Data Manager. Researchers access the treatment allocation for each participant by means of a remote, internet-based randomisation system developed and maintained by the Nottingham CTU. The sequence of treatment allocations will be concealed until interventions have all been assigned and recruitment, data collection, and all other trial-related assessments are completed. The trial is single-blind, with participants and local investigators administering the interventions being aware of the treatment allocation. Outcome measures are administered in a blinded fashion by researchers to minimise the effects of lack of blinding.

### Interventions

Psycho-education combined with problem solving (PEPS) therapy is a complex cognitive-behavioural intervention that integrates individual and group therapies. Psycho-education is an individual 4-session collaborative dialogue designed to build a rapport with patients, inform them about their personality disorder, discuss its effects on interpersonal relationships and social functioning, and enhance motivation for therapy [21]. In psycho-education, participants are taken through their personality disorder diagnoses, as identified via a structured clinical assessment. Participants are asked what problems they experience in relation to their personality disorder and they are then guided to specify problems which are then prioritised to be addressed in the problem solving therapy sessions.

Problem solving therapy is a 12-session group intervention designed to teach people strategies for solving interpersonal problems [9]. Participants are encouraged to learn the process of a) identifying negative feelings and using these as a cue for initiating the problem solving process; b) defining their problem clearly and accurately; c) setting specific goals for change; d) generating solution options; e) considering the consequences of each option; and f) selecting potentially effective options and organising these into a means-end action plan. Participants are then expected to implement the action plan and are offered individual support sessions to help with implementation. Progress with the action plan is reviewed in the next group session.

### Assessments

#### Screening

Screening for personality disorder is via the International Personality Disorder Examination (IPDE) [27]. This is a 99-item, semi-structured interview that allows both diagnostic and dimensional scores to be extracted for each personality disorder according to either DSM or ICD criteria.

#### Primary outcome

The primary outcome is social functioning, measured by the Social Functioning Questionnaire (SFQ) [23]. The SFQ is an 8-item self-report scale, with items covering the domains of home, work, leisure, and relationships. Respondents rate the extent to which they have experienced problems in each area over the last two weeks on a scale from 0 to 3. SFQ scores correlate well with measures of psychiatric distress and are stable over time. A reduction (i.e., an improvement) of 2 points or more on the SFQ at follow up 72 weeks post-randomisation is our specified clinically significant change.

#### Secondary outcomes

Measures of a number of secondary outcomes are listed below.

##### Receipt and cost of services

(Client Service Receipt Inventory; CSRI [31]). One measure used to capture service use is the CSRI, which records health and social care, criminal justice, informal care services, employment and benefits as reported by the participant. This information will be used in the cost-effectiveness analysis. Service use will be assessed for the period 6 months prior to baseline, and at three points after treatment (immediately after, at 4 months and at 72 weeks). The long-term follow up is necessary to pick up potential improvement overall, rather than capture a temporary increase at the end of treatment.

##### Scheduled and unscheduled service use (Record Check)

People with PD are often long-term users of services, but they can be chaotic users of those services. Hence, one valuable outcome would be a more systematic use of the services available. We hope to find a reduction of unscheduled service usage and an increase in scheduled service usage. Data on mental health service use, Emergency Department attendances and hospital admissions will be collected through a review of mental health service and GP records to ascertain use of scheduled and unscheduled services. Service use data will be collected retrospectively for the duration of involvement in the trial, from baseline to 72 week follow-up.

##### Quality of life

(EuroQOL; EQ-5D) [32]. The EQ-5D is a health-related quality of life measure and will be used to generate quality-adjusted life years for use in the economic evaluation. The EQ-5D will be administered before and after treatment, and again at 72 week follow-up.

##### Referrer's assessment of problems

(change in referrer's score on Global Assessment of Functioning; GAF) [33]. The GAF is the standard method for representing a clinician's judgment of a patient's overall level of psychosocial functioning and will be rated by the referrer.

##### Anxiety and depression

(Hospital Anxiety and Depression Scale; HADS) [30]. A reduction in mental distress is an important outcome for service users [17], therefore anxiety and depression will be measured using the 14-item HADS.

##### Client's assessment of problems

(i.e., specific treatment targets for individuals). A focus on the problems most relevant to the client is considered important [34]. Participants will be asked to identify their three most important problems and rate their severity before and after treatment.

#### Intermediate outcomes

Two intermediate outcomes are assessed.

##### Treatment alliance

(Working Alliance Inventory; WAI) [35]. The WAI examines the development of treatment alliance, and will be used to assess the effectiveness of the psycho-education component in developing treatment alliance. The WAI is a 36-item questionnaire that can be administered to both clients and therapists. Each item is rated on a 7-point scale and scores are produced on three factors: the therapeutic bond, the agreement on goals, and the agreement on tasks.

##### Social problem solving skills

(Social Problem Solving Inventory-Revised; SPSI-R) [13]. The SPSI-R assesses the development of social problem solving skills to examine whether the social problem solving component improves these skills as expected. The SPSI-R is a 25-item client self-report questionnaire that measures problem solving orientation (positive and negative) and problem solving style (rational, impulsive and avoidant).

#### Process measures

Information will be gathered regarding the route of referral, sessions offered, and participants' attendance. Semi-structured interviews will access information about experiences of therapy, its perceived effects and its perceived limitations. Interviews will also provide an opportunity to support people after psycho-education, since some participants report finding this distressing [36].

**Figure 2:**
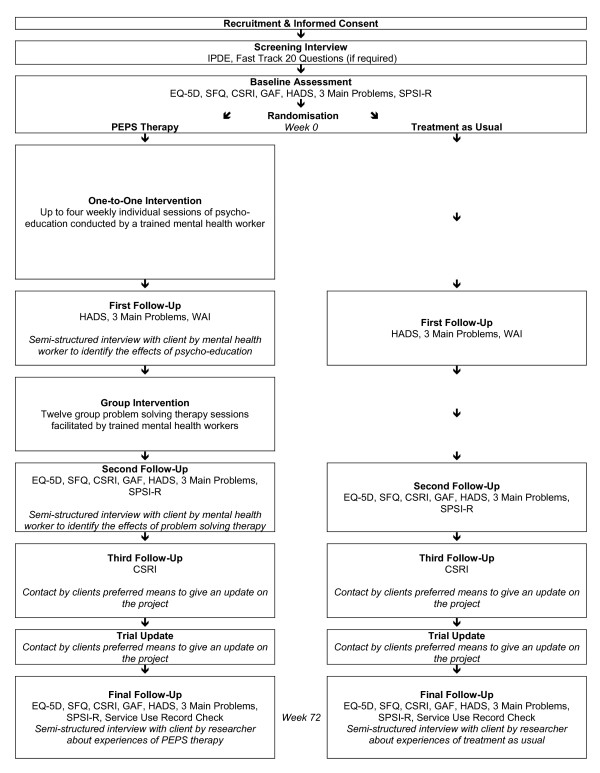
**Procedural Flow Diagram**.

A flow chart of the procedures in the PEPS trial is presented in Figure 2.

### Analyses

Demographic and other baseline data will be summarised by descriptive statistics (number [N], mean, standard deviation [SD], median, minimum and maximum) or frequency tables, stratified by treatment. Measures of adherence to protocol implementation will be summarised by descriptive statistics (number [N], mean, standard deviation [SD], median, minimum and maximum) or frequency tables, stratified by treatment arm. Efficacy will be assessed on the full analysis set, defined as all randomised participants for whom a post-baseline assessment of the primary endpoint is available, that is, in accordance with the "intention to treat" (ITT) principle. The primary efficacy parameter will be the difference in mean SFQ score between treatment arms, estimated using a hierarchical regression model with adjustment for relevant covariates, including centre, sex and personality disorder classification. Differences in mean totals of secondary outcomes at 72 weeks from randomisation will be compared between the two treatment arms, after adjusting for design (centre and sex) covariates. Analyses will be performed using Stata version 11 or above.

The costs of the interventions will be estimated by combining data on number of sessions provided with unit costs derived from local data on service expenditure and activity. These costs will include therapist time, oncosts, overheads and capital. Costs of other services will be calculated by combining service use data collected with the CSRI with appropriate unit costs. Costs will be compared between the two groups, with bootstrap methods used to generate confidence interval round the difference due to the expected skewed cost distribution. Cost-effectiveness will be assessed by combining the cost adapt with outcomes, namely SFQ scores and Quality-Adjusted Life Years (QALYs). If the costs for the PEPS arm are less than for TAU and the outcome better, then it will be the 'dominant' intervention. However, if costs are greater and outcomes better, then an incremental cost-effectiveness ratio will indicate the extra costs incurred to achieve an extra unit-improvement in outcome (i.e., a one-point improvement on the SFQ or one extra QALY). There will be uncertainty around the point estimates of cost and outcome differences and this uncertainty will be explored by generating a large number of cost-outcome combinations using bootstrapped resample and plotting these on a cost-effectiveness plane. Cost-effectiveness will be interpreted using cost-effectiveness acceptability curves. These will show the probability that PEPS is more cost-effective intervention than TAU for a range of values placed on a unit-improvement in outcome. The range of values for a one-point improvement on the SFQ is unclear, but will be chosen such that we will be able to observe the points at which there is at least at 50% and 80% probability of PEPS being cost-effective. The range of values for a gain of one QALY is more straightforward as it appears that the value that informs NICE recommendations is between £20-30K. Therefore we will use a range of £0-£100K to produce the curve so as to include this threshold.

Thematic analysis methods will be used to specify the nature of treatment-as-usual in terms of what problem was treated by which service/professional and how. Thematic analysis methods will be used to identify themes in participants' opinions of PEPS therapy and TAU.

## Discussion

PEPS therapy has potential as an economical, accessible, and acceptable intervention for people with personality disorder. Individual psychoeducation has the potential to enhance engagement in the group therapy that follows [21,25], thus addressing the important issue of treatment non-completion [19,20]. Problem solving therapy, a major component of PEPS, has a good track record with a range of physical health and mental health problems [14-16], but its effectiveness has not yet been examined with personality disorder. Referring to the PEPS pilot study [22], one commentator noted that the relatively brief PEPS intervention challenges the notion that long-term treatment is always necessary for PD: "The idea that people with personality disorders can only improve after years of treatment has led to a relative neglect of this patient group, based on the belief that they cannot benefit from pragmatic treatment. This study, along with several others, shows that significant progress can be made with less expensive and briefer interventions" [26, p. 121]. There is a recent drive to evaluate briefer interventions for PD [37]. Another commentator noted that, if the PEPS intervention proves cost-effective, then this would "not only improve the evidence base for treatment of personality disorder, but [...] might also go some way towards challenging the ambivalence that some healthcare professionals continue to have about working with people with personality disorders" [25, p. 284]. The PEPS intervention does, therefore, have positive potential in a number of ways.

There are, however, challenges to be met in the course of conducting the trial. We face the potential problem of drop-out between consent to participate and the start of group therapy, since there will be delays caused by waiting for sufficient numbers to accrue in order to start group treatment. This problem will be exacerbated by the intensive nature of the assessment procedure which participants will need to undertake before randomization, hence slowing down the accrual of participants ready for randomization.

There are also limitations that need to be acknowledged. First, this is not a double-blind study design and so the participants and some of the researchers collecting interim follow-up information will be aware of participant allocation. However, the researchers and statisticians collecting and analysing the follow-up data will be blind to treatment status. The risk of unblinding researchers will be minimised by instructing participants and clinical teams not to disclose treatment details at any stage. Cases of possible unblinding of researchers will be logged. Second, although this is a pragmatic trial, there are extensive assessments, necessary for research purposes, that would likely not be conducted in actual clinical practice. These assessments may have an impact on outcome, even without the active treatment that follows in the treatment arm. This may minimise differences between arms.

If we overcome potential difficulties and minimise limitations, the results from this randomised controlled trial will tell us if PEPS therapy is effective and cost-effective. If it proves to be so, then it will be a useful treatment for inclusion in a broader menu of treatment options for this group of service users.

## Competing interests

The authors declare that they have no competing interests.

## Authors' contributions

MM, HW, MJC, PMcC and CEA contributed to the design of the study. All authors contributed to the creation of the Manual of Procedures and the study protocol. MM drafted the manuscript. All authors provided a critical review and final approval of the manuscript.
